# Supplementary material for: Lifestage Sex-Specific Genetic Effects on Metabolic Disorders in an Adult Population in Korea: The Korean Genome and Epidemiology Study
Source: Int J Mol Sci. 2022 Oct 6;23(19):11889. doi: 10.3390/ijms231911889 (PMC9569480; doi:10.3390/ijms231911889)
Supplement: Supplementary file 1 [file ijms-23-11889-s001.zip › Supplementary Figure S9. Signal plots for each sig locus.pptx]

## Slide 1
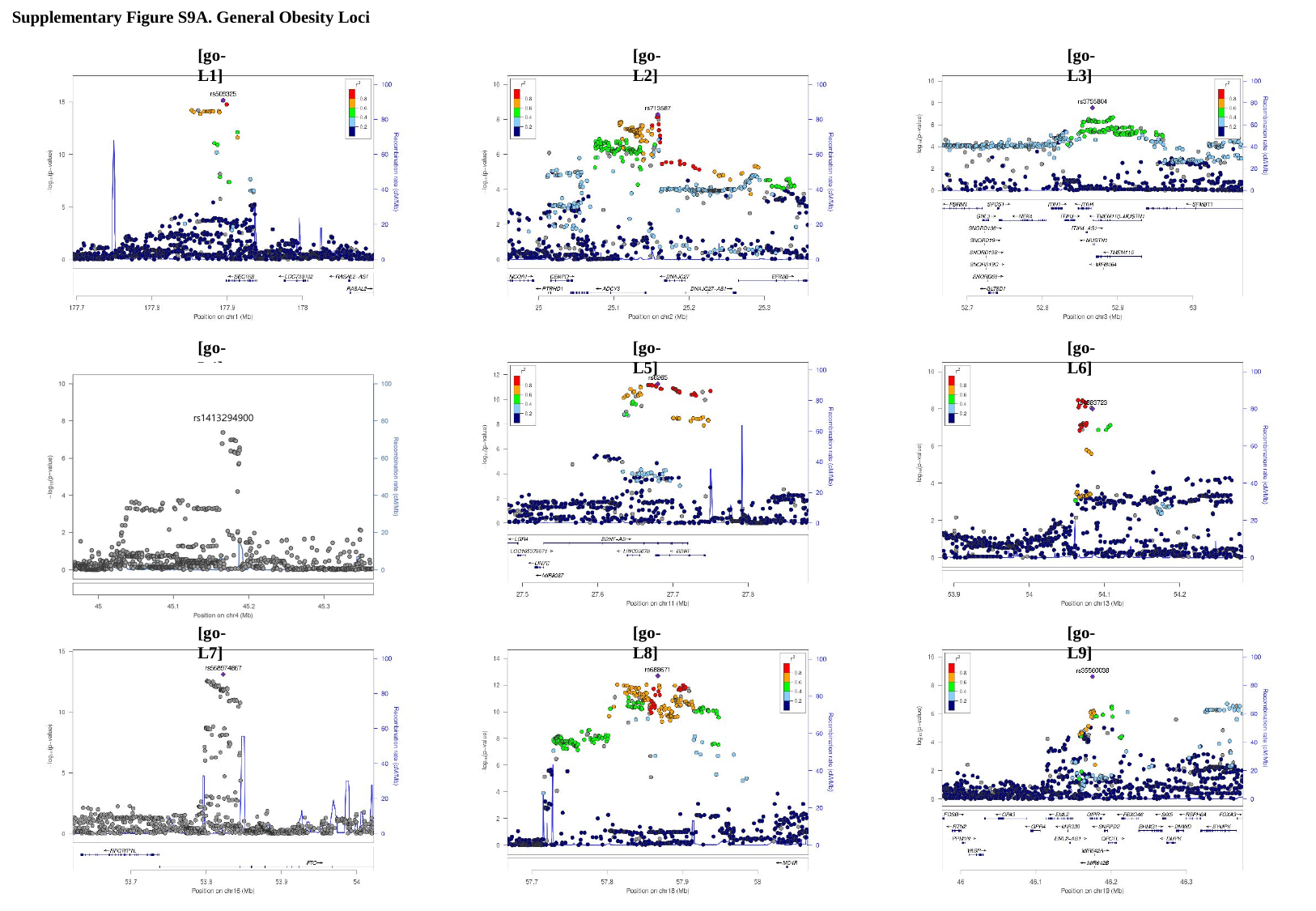

Supplementary Figure S9A. General Obesity Loci
[go-L1]
[go-L2]
[go-L3]
[go-L4]
[go-L5]
[go-L6]
[go-L7]
[go-L8]
[go-L9]

## Slide 2
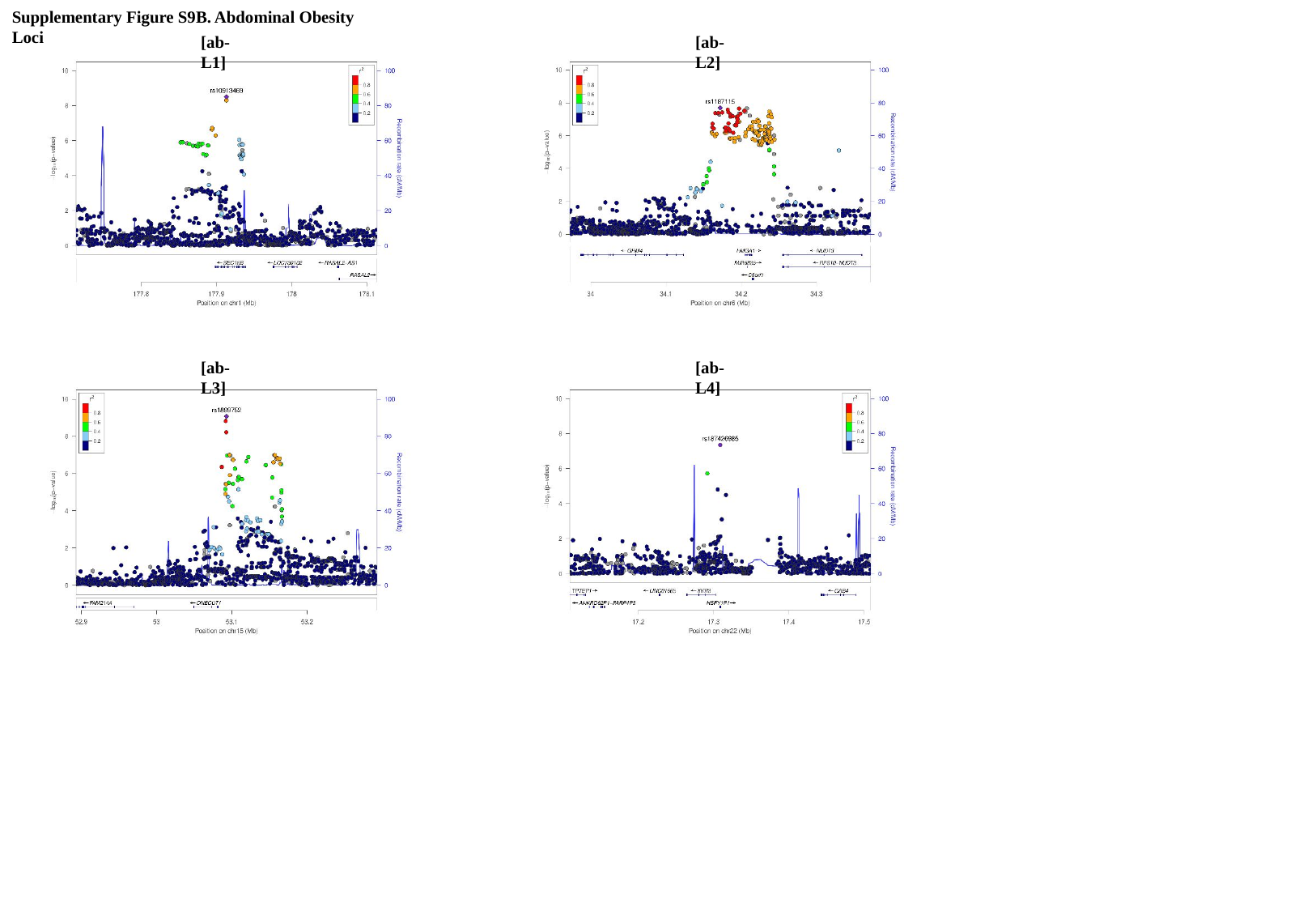

Supplementary Figure S9B. Abdominal Obesity Loci
[ab-L1]
[ab-L2]
[ab-L3]
[ab-L4]

## Slide 3
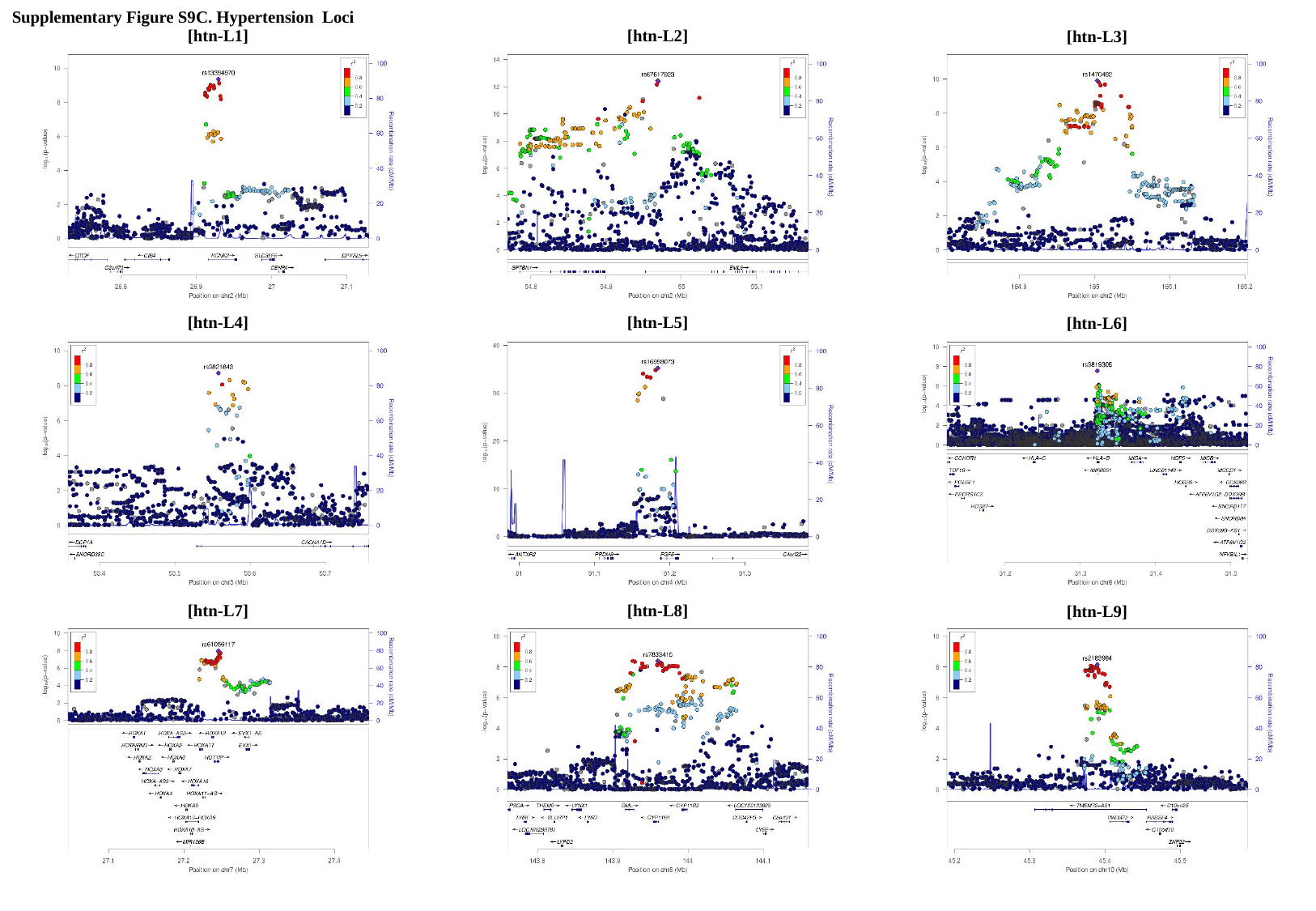

Supplementary Figure S9C. Hypertension Loci
[htn-L1]
[htn-L2]
[htn-L3]
[htn-L4]
[htn-L5]
[htn-L6]
[htn-L7]
[htn-L8]
[htn-L9]

## Slide 4
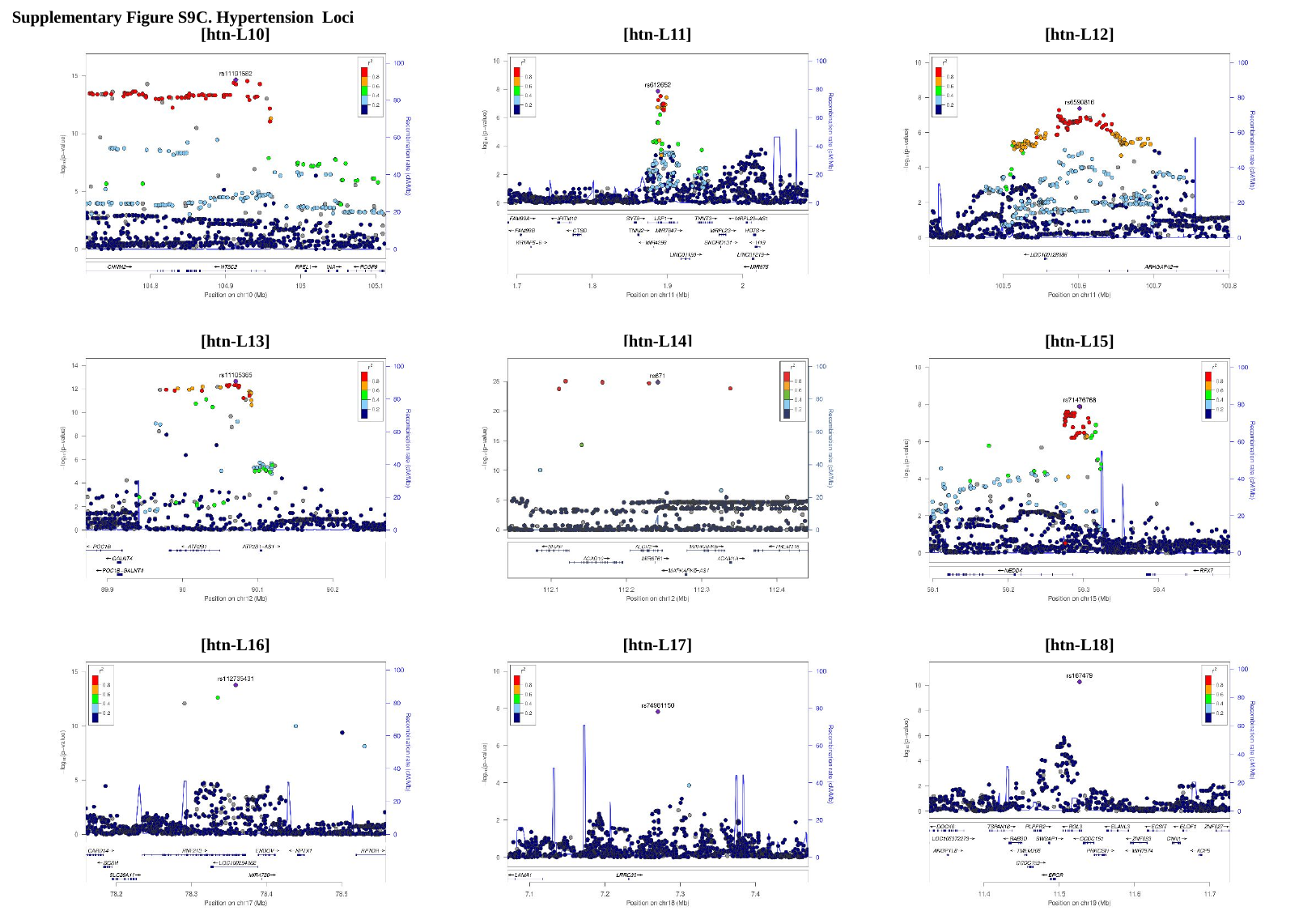

Supplementary Figure S9C. Hypertension Loci
[htn-L10]
[htn-L11]
[htn-L12]
[htn-L13]
[htn-L14]
[htn-L15]
[htn-L16]
[htn-L17]
[htn-L18]

## Slide 5
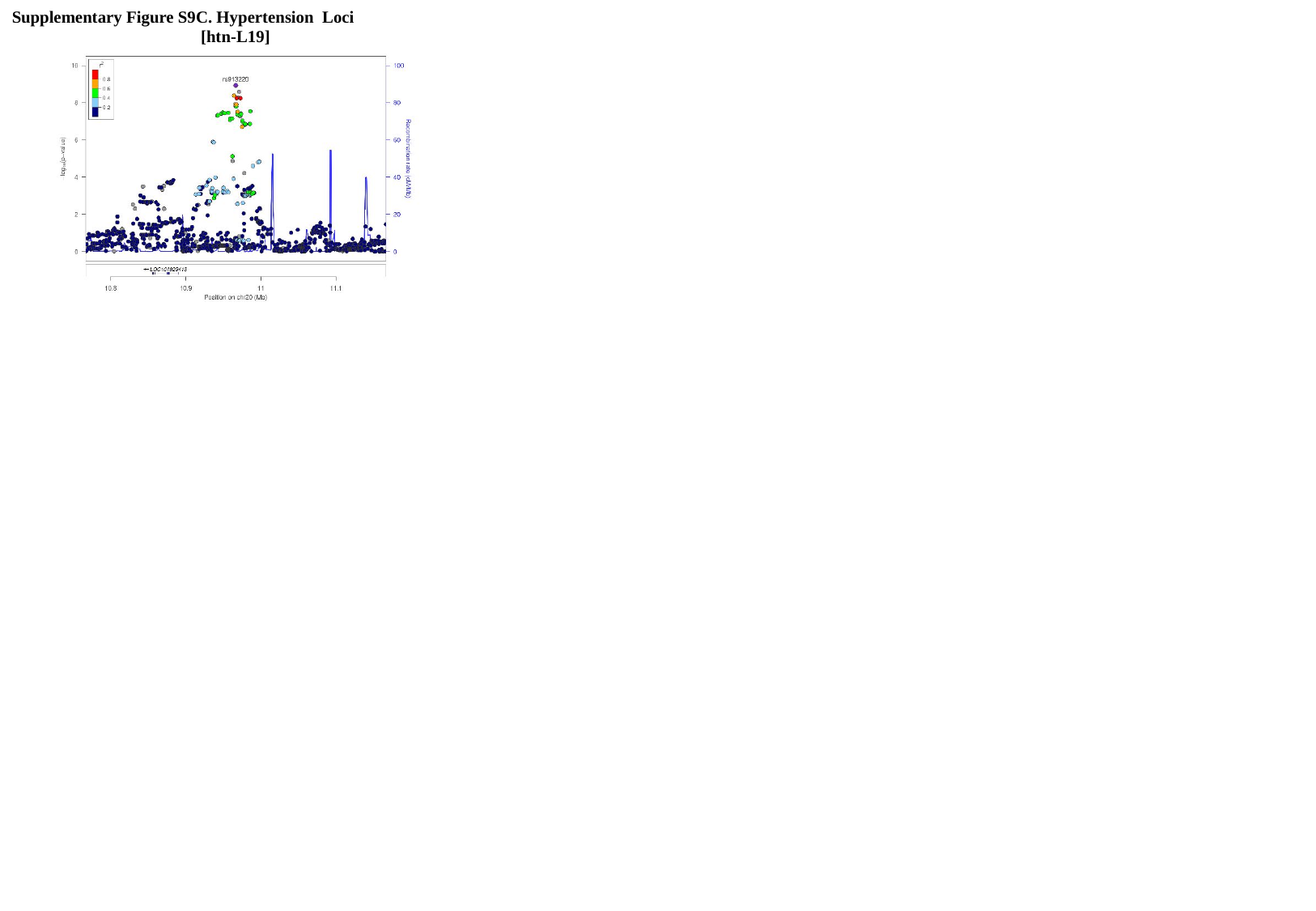

Supplementary Figure S9C. Hypertension Loci
[htn-L19]

## Slide 6
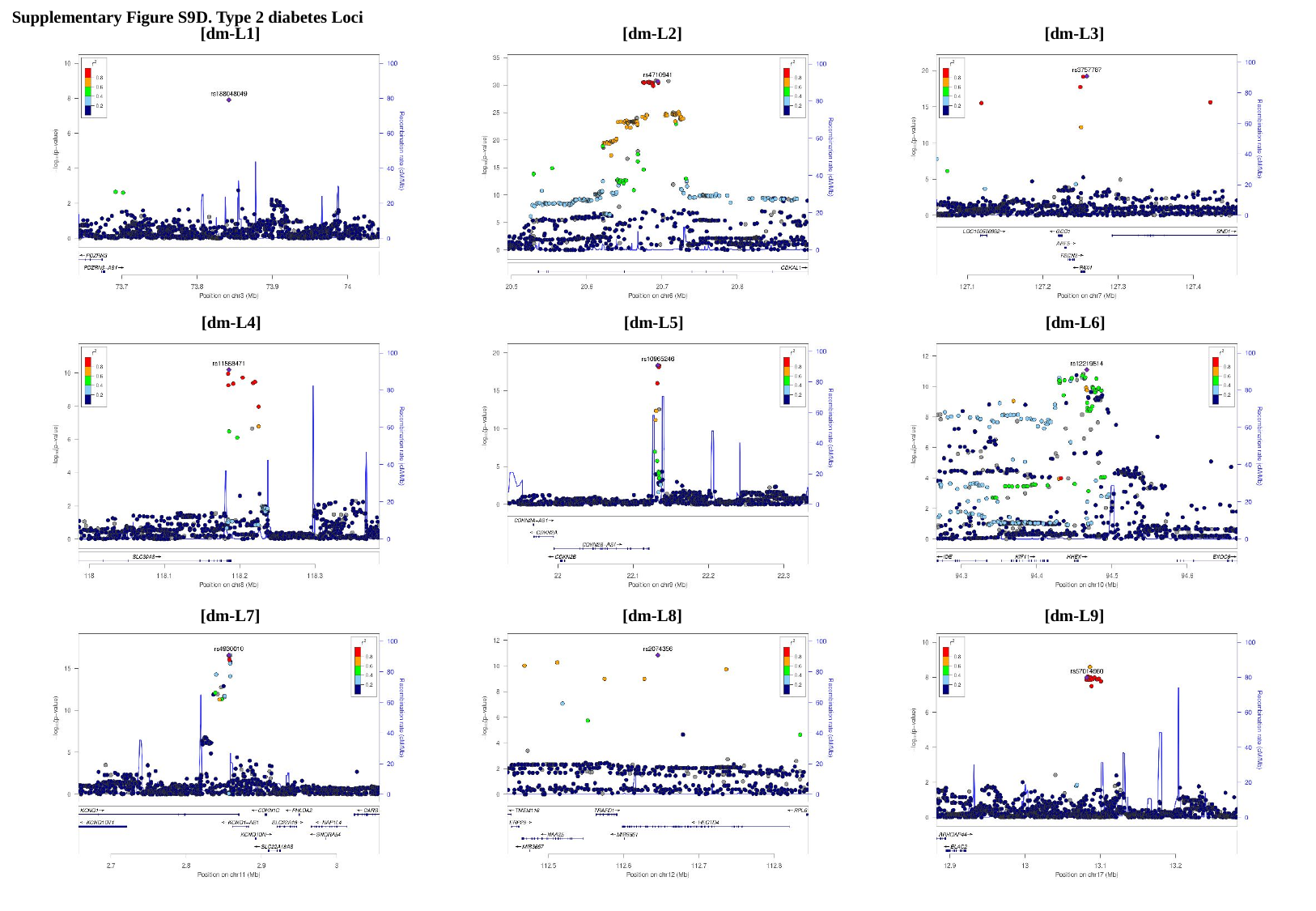

Supplementary Figure S9D. Type 2 diabetes Loci
[dm-L1]
[dm-L2]
[dm-L3]
[dm-L4]
[dm-L5]
[dm-L6]
[dm-L7]
[dm-L8]
[dm-L9]

## Slide 7
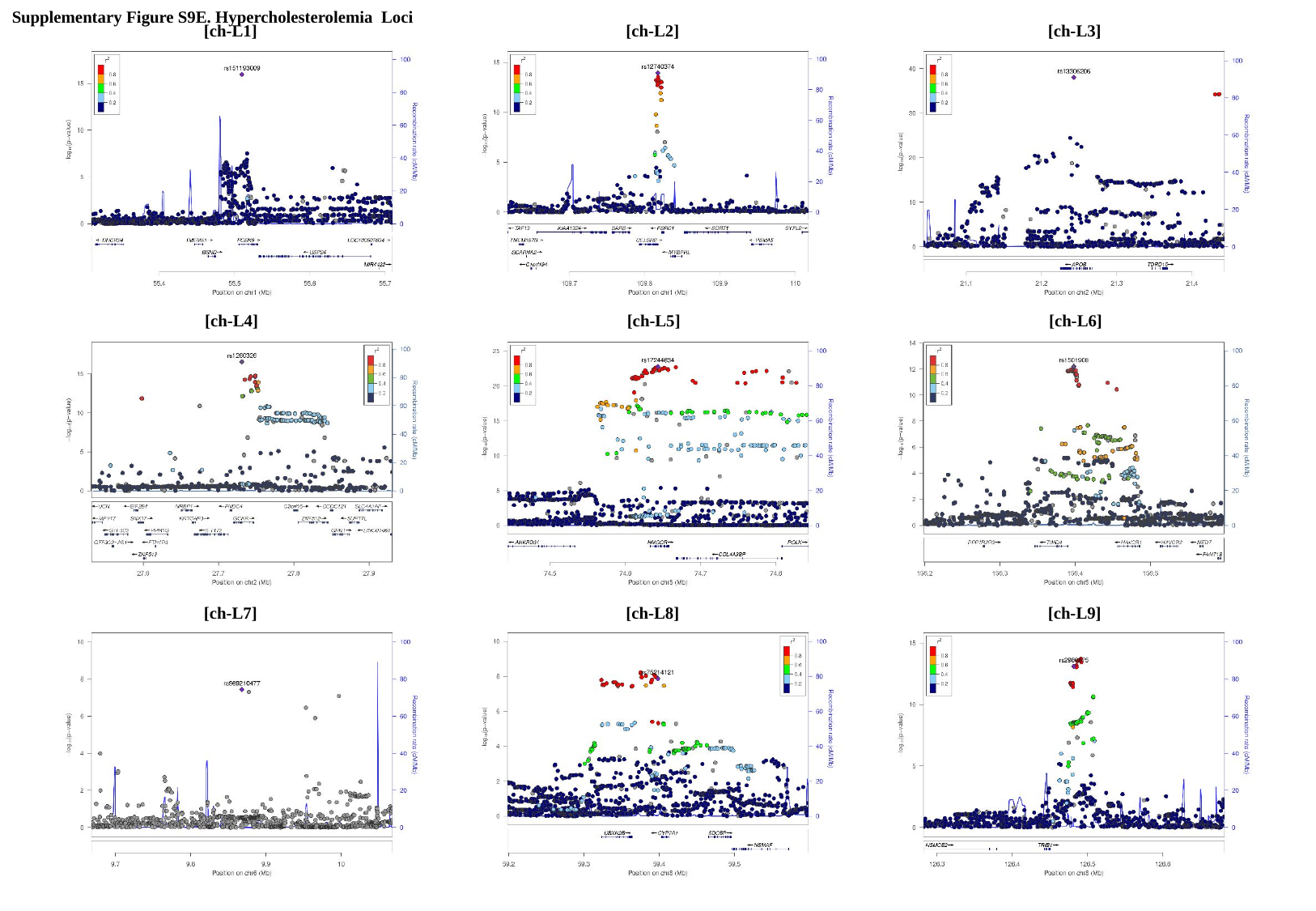

Supplementary Figure S9E. Hypercholesterolemia Loci
[ch-L1]
[ch-L2]
[ch-L3]
[ch-L4]
[ch-L5]
[ch-L6]
[ch-L7]
[ch-L8]
[ch-L9]

## Slide 8
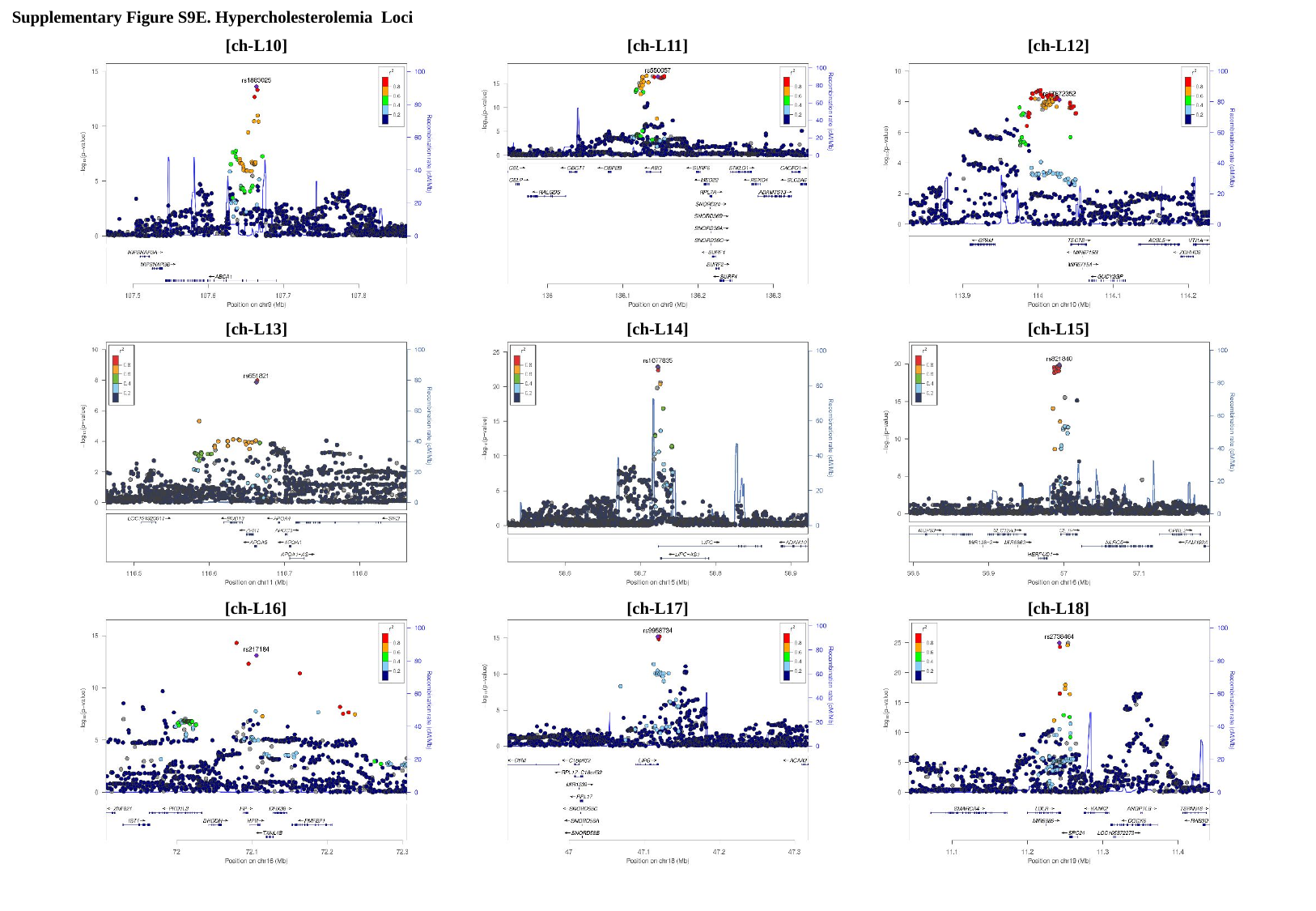

Supplementary Figure S9E. Hypercholesterolemia Loci
[ch-L10]
[ch-L11]
[ch-L12]
[ch-L13]
[ch-L14]
[ch-L15]
[ch-L16]
[ch-L17]
[ch-L18]

## Slide 9
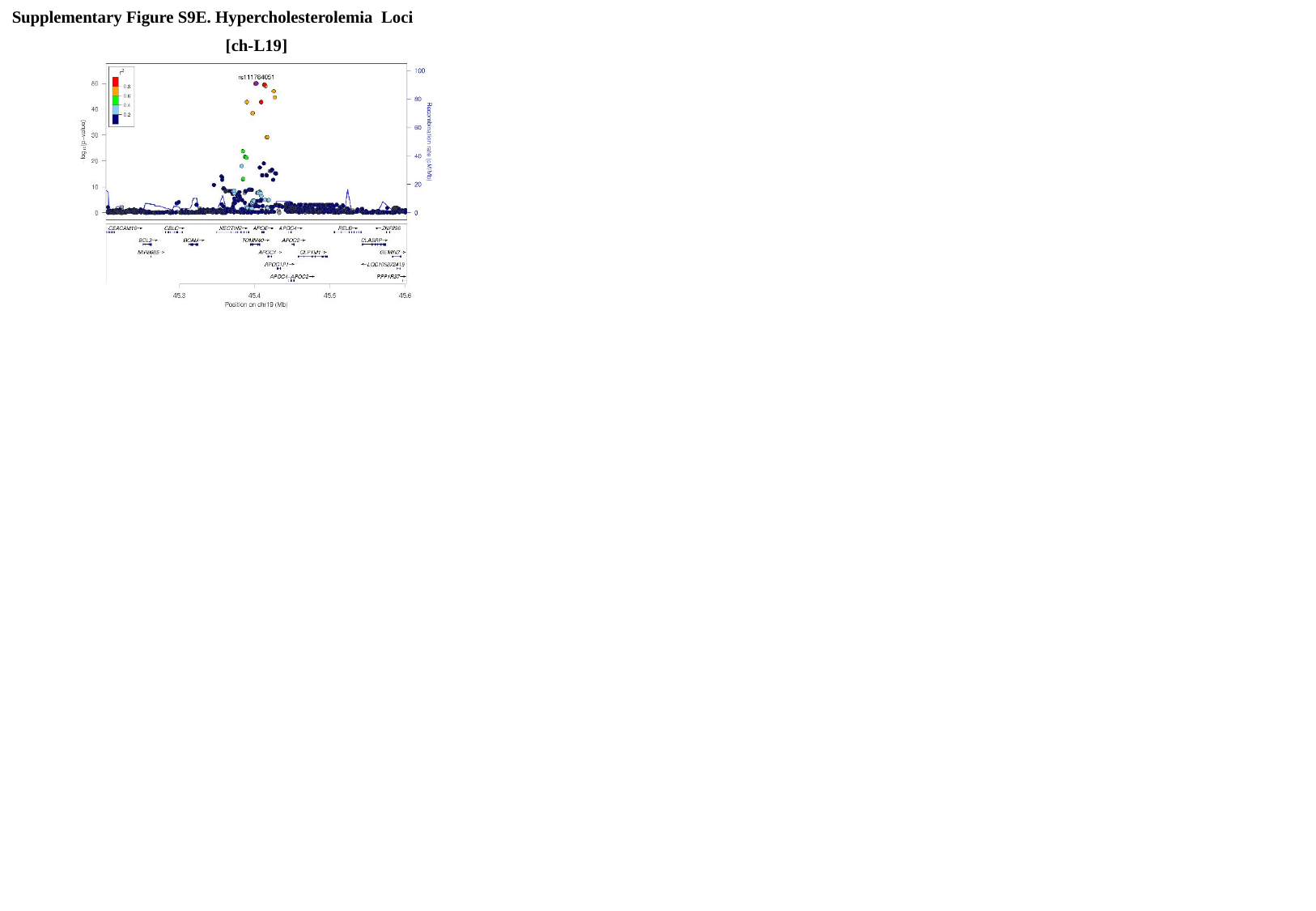

Supplementary Figure S9E. Hypercholesterolemia Loci
[ch-L19]

## Slide 10
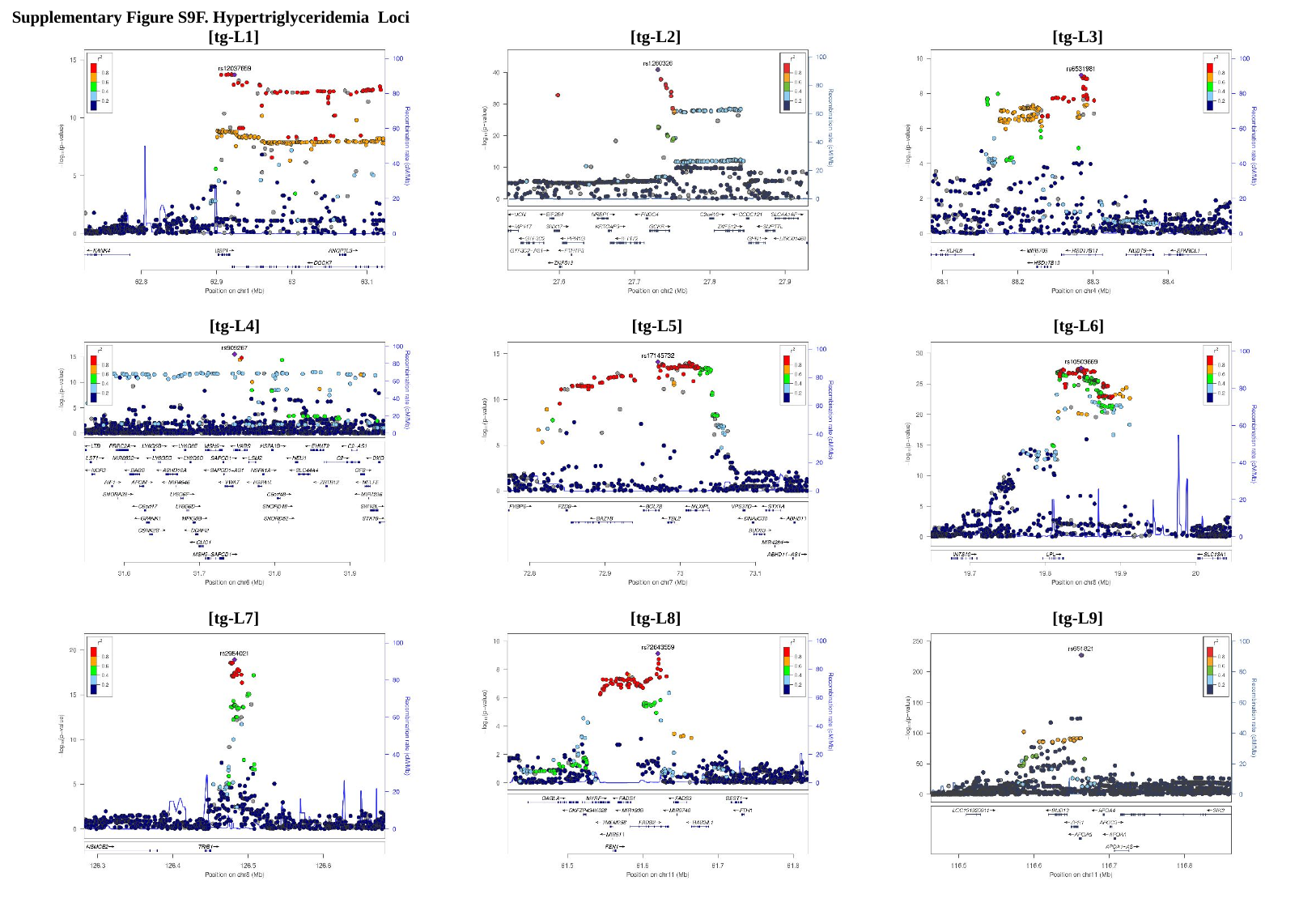

Supplementary Figure S9F. Hypertriglyceridemia Loci
[tg-L1]
[tg-L2]
[tg-L3]
[tg-L4]
[tg-L5]
[tg-L6]
[tg-L7]
[tg-L8]
[tg-L9]

## Slide 11
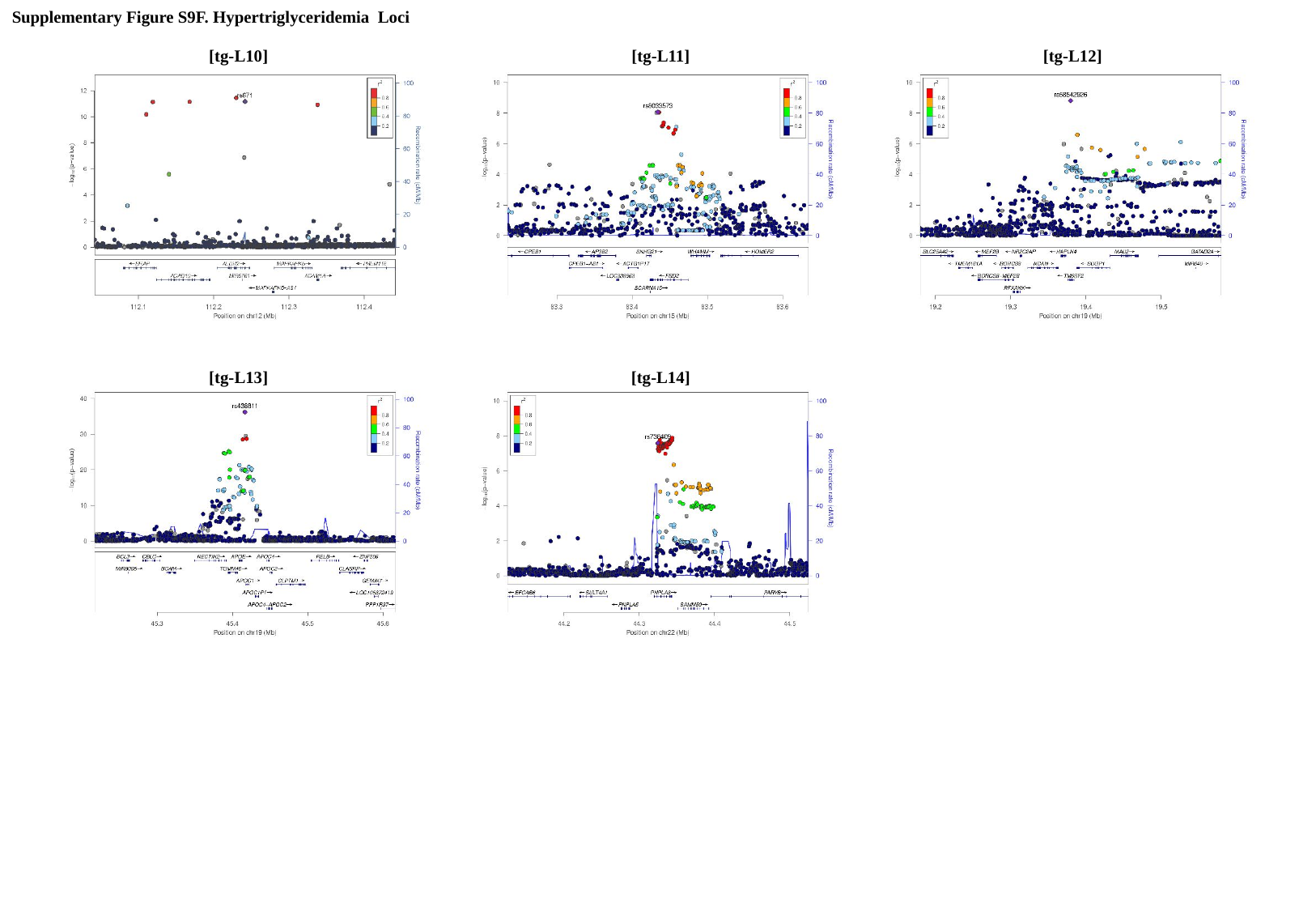

Supplementary Figure S9F. Hypertriglyceridemia Loci
[tg-L10]
[tg-L11]
[tg-L12]
[tg-L13]
[tg-L14]

## Slide 12
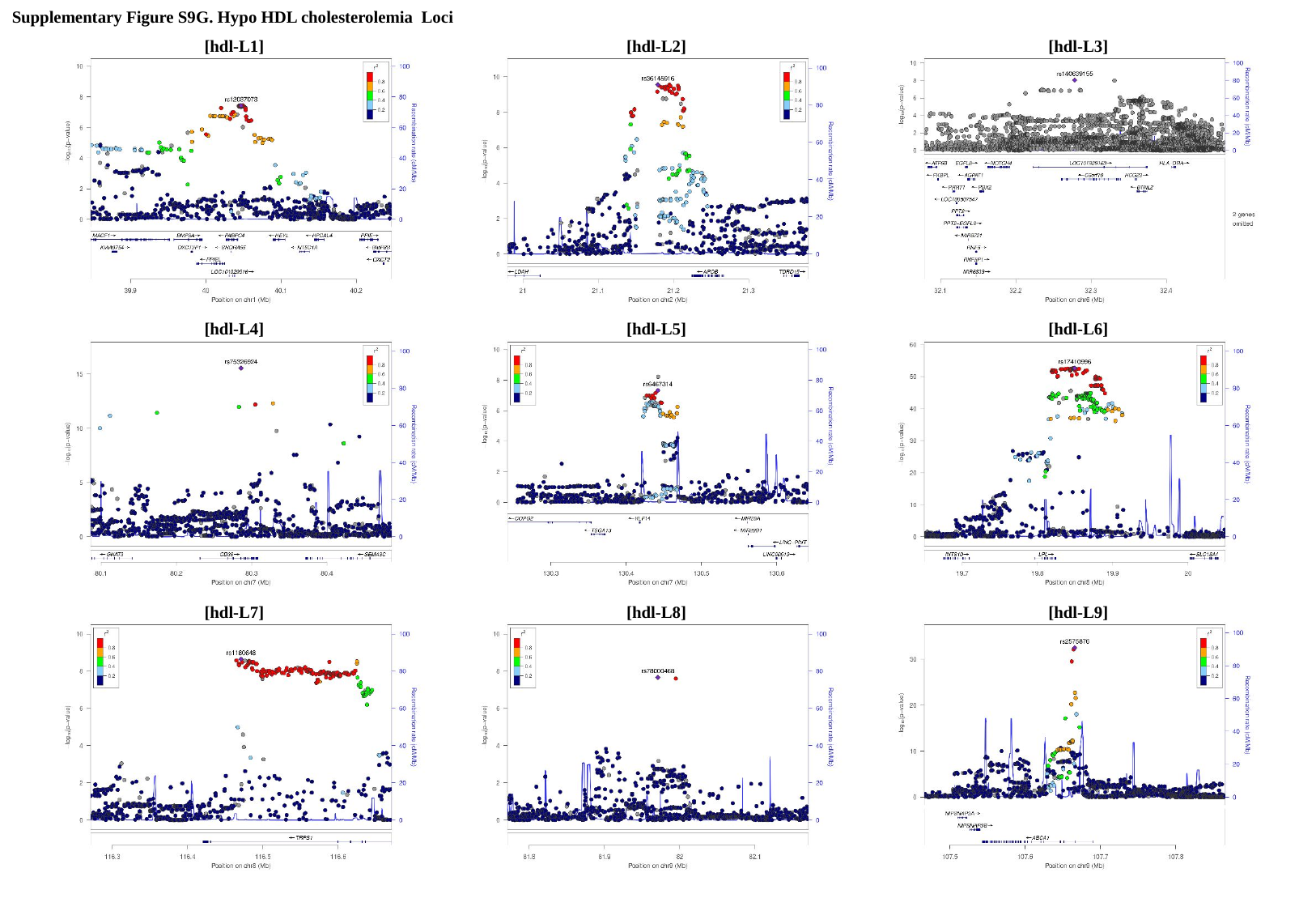

Supplementary Figure S9G. Hypo HDL cholesterolemia Loci
[hdl-L1]
[hdl-L2]
[hdl-L3]
[hdl-L4]
[hdl-L5]
[hdl-L6]
[hdl-L7]
[hdl-L8]
[hdl-L9]

## Slide 13
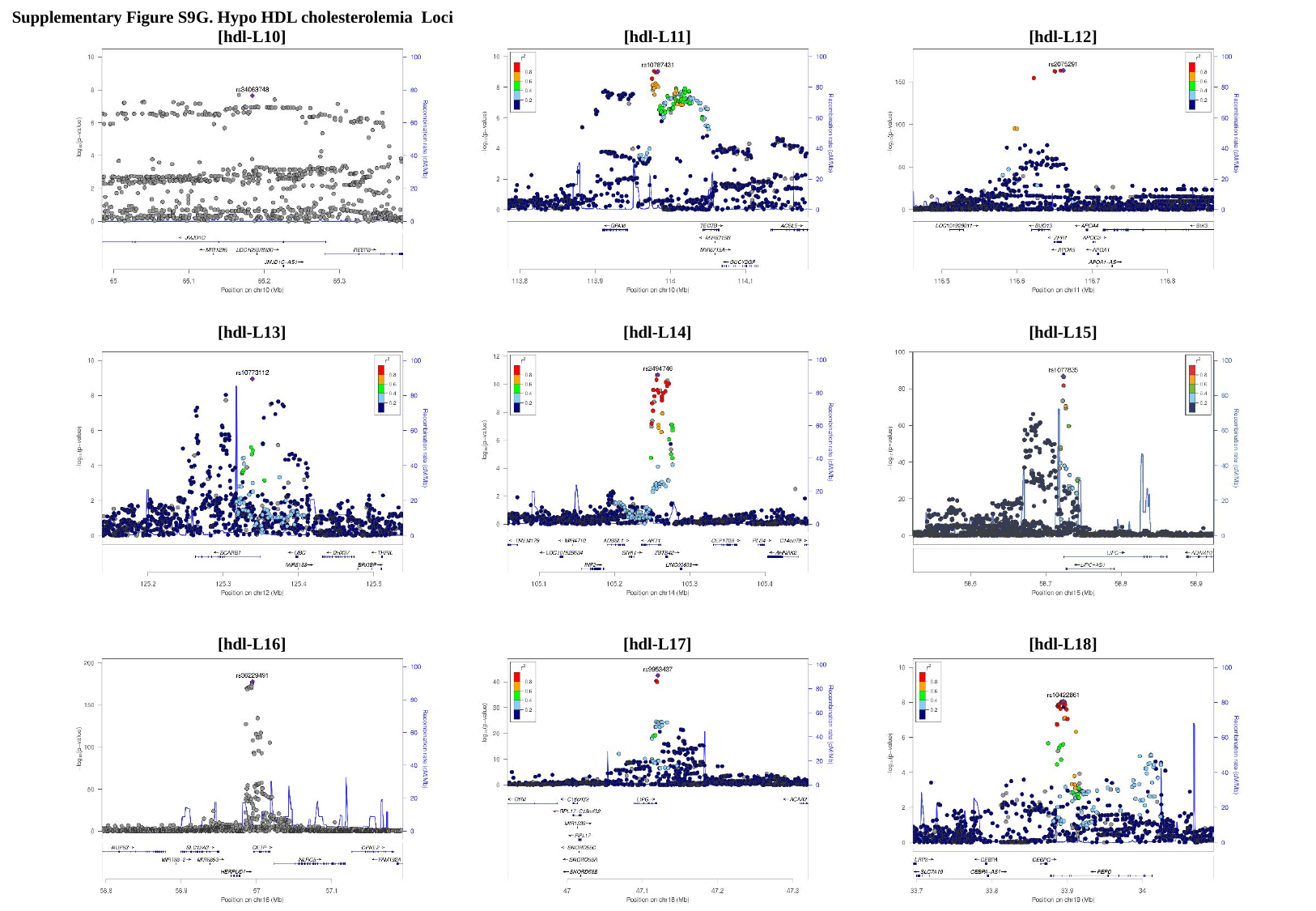

Supplementary Figure S9G. Hypo HDL cholesterolemia Loci
[hdl-L10]
[hdl-L11]
[hdl-L12]
[hdl-L13]
[hdl-L14]
[hdl-L15]
[hdl-L16]
[hdl-L17]
[hdl-L18]

## Slide 14
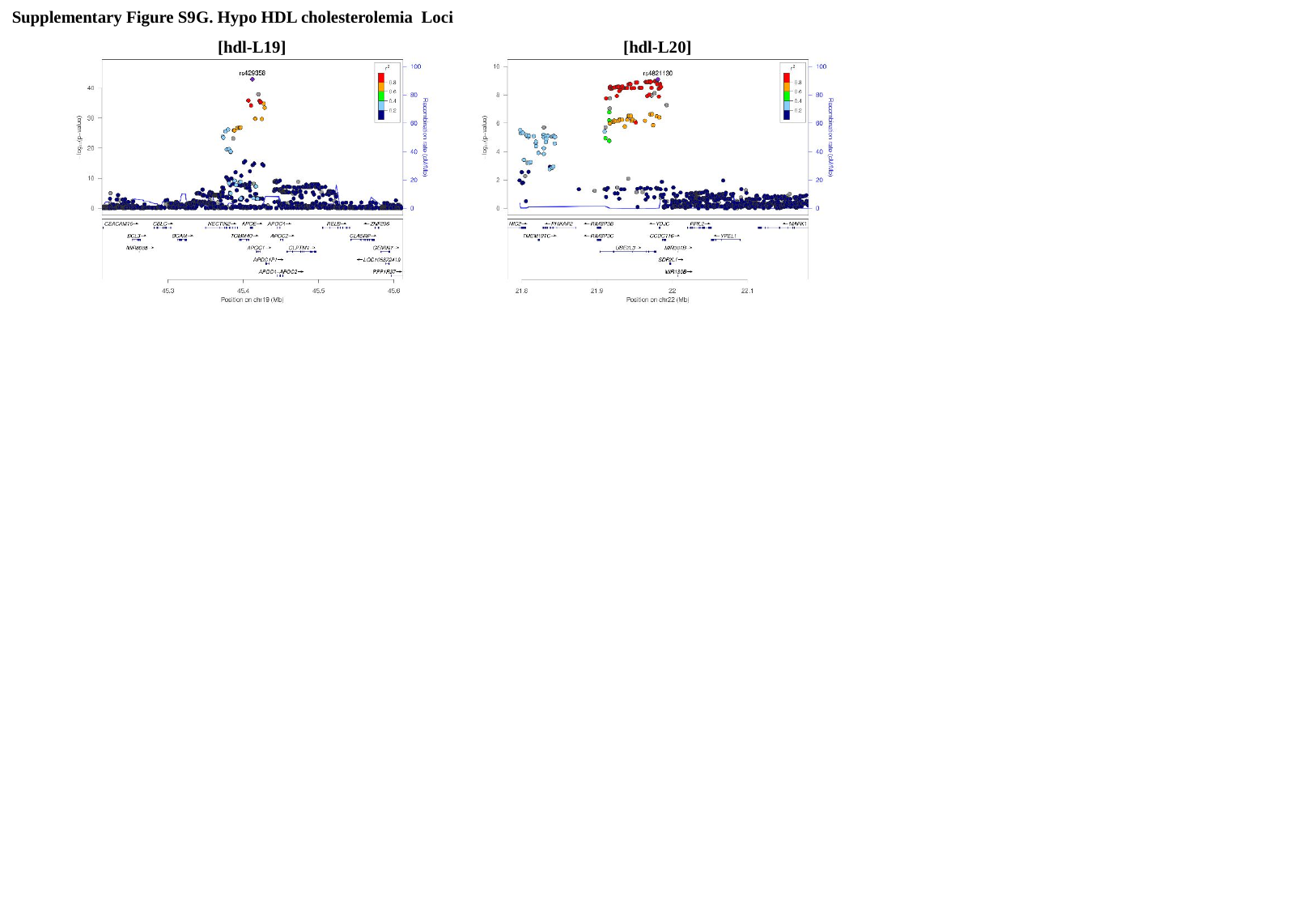

Supplementary Figure S9G. Hypo HDL cholesterolemia Loci
[hdl-L19]
[hdl-L20]

## Slide 15
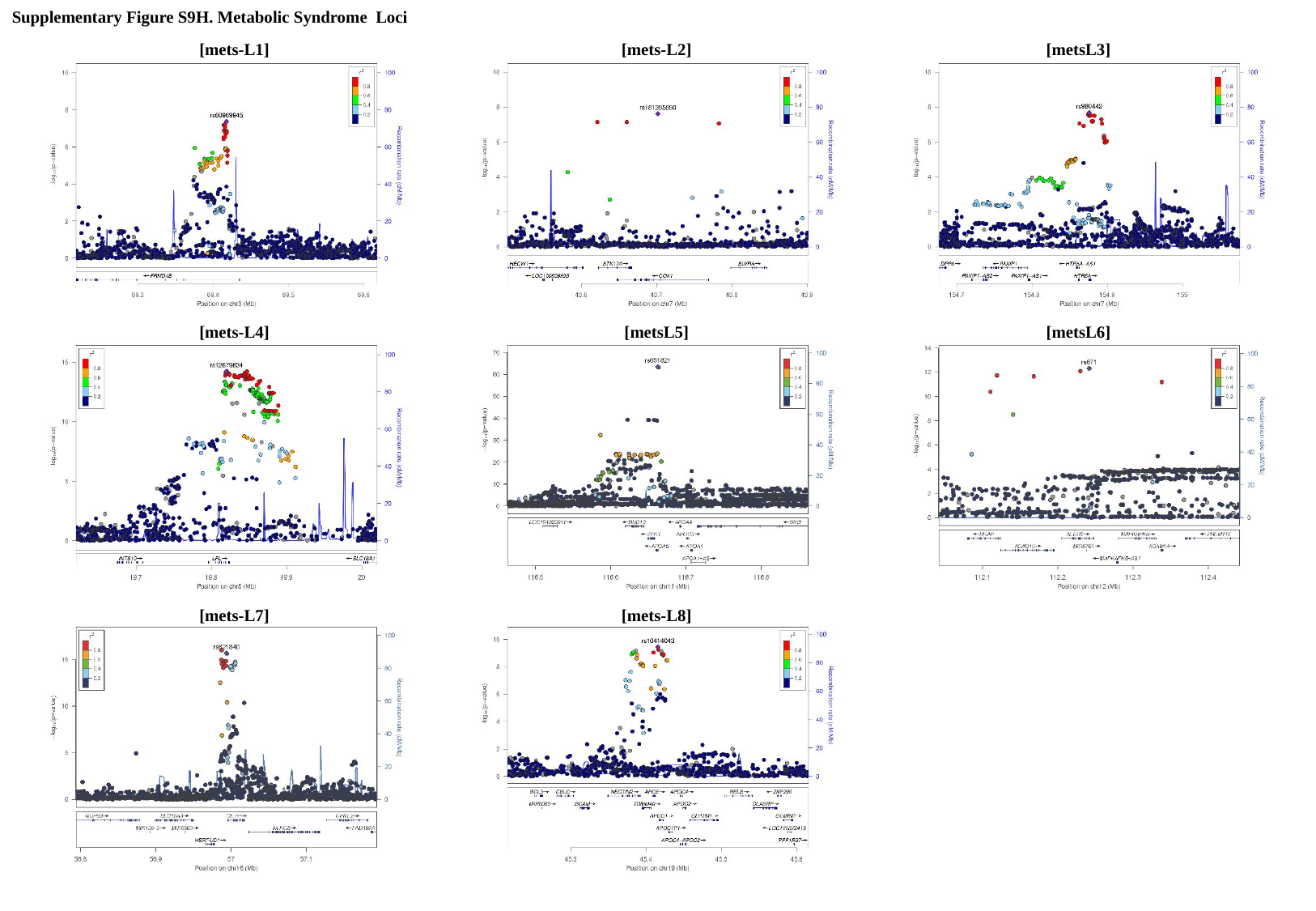

Supplementary Figure S9H. Metabolic Syndrome Loci
[mets-L1]
[mets-L2]
[metsL3]
[mets-L4]
[metsL5]
[metsL6]
[mets-L7]
[mets-L8]
