# Supplementary figures and images for: Lifestage Sex-Specific Genetic Effects on Metabolic Disorders in an Adult Population in Korea: The Korean Genome and Epidemiology Study
Source: Int J Mol Sci. 2022 Oct 6;23(19):11889. doi: 10.3390/ijms231911889 (PMC9569480; doi:10.3390/ijms231911889)

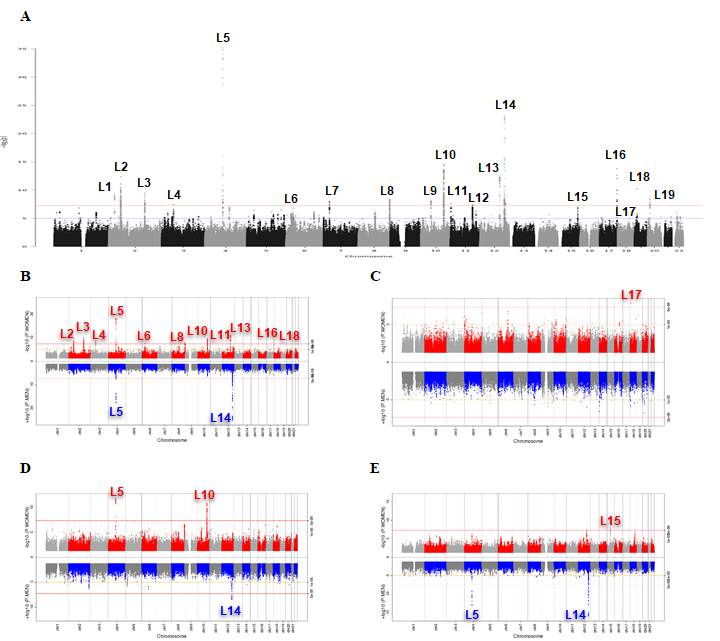

Supplement: Supplementary file 1 [file ijms-23-11889-s001.zip › Supplementary Figure S3. Manhattan & Maimi plot on HTN.tif]

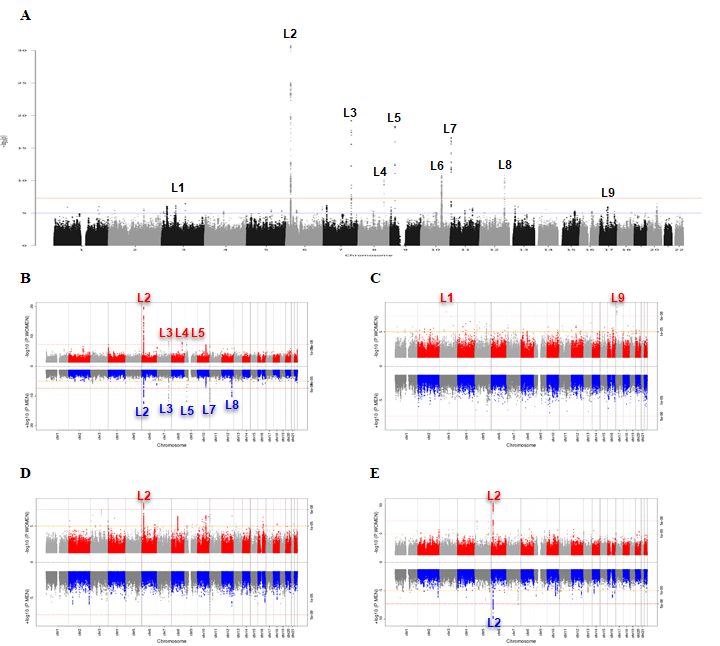

Supplement: Supplementary file 1 [file ijms-23-11889-s001.zip › Supplementary Figure S4. Manhattan & Maimi plot on T2DM.tif]

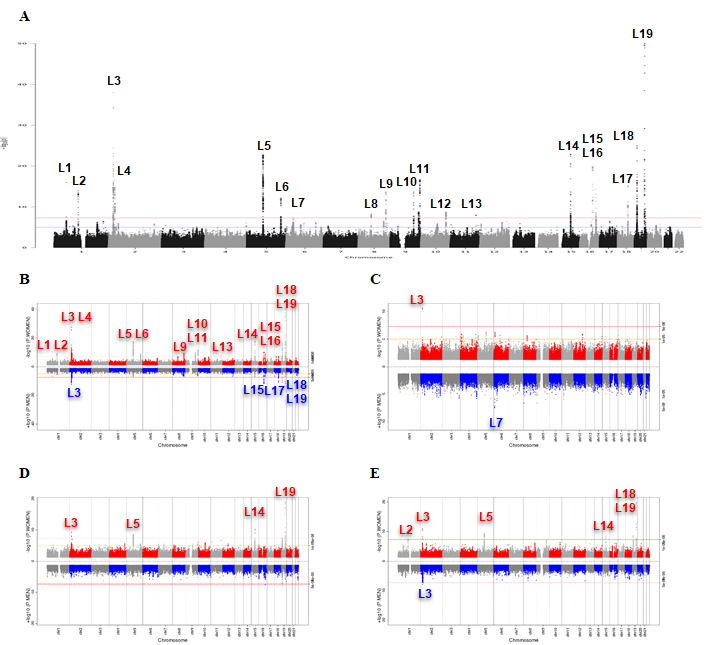

Supplement: Supplementary file 1 [file ijms-23-11889-s001.zip › Supplementary Figure S5. Manhattan & Maimi plot on TCHL.tif]

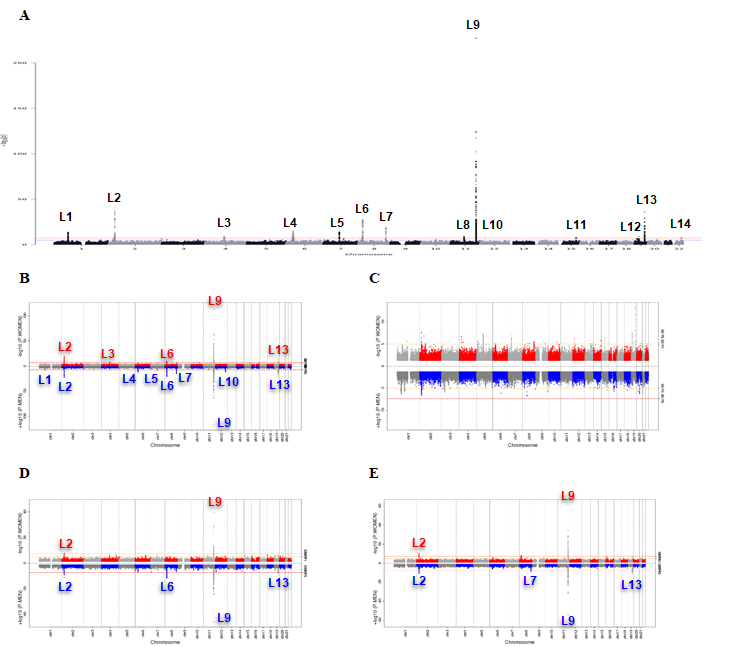

Supplement: Supplementary file 1 [file ijms-23-11889-s001.zip › Supplementary Figure S6. Manhattan & Maimi plot on TG.tif]

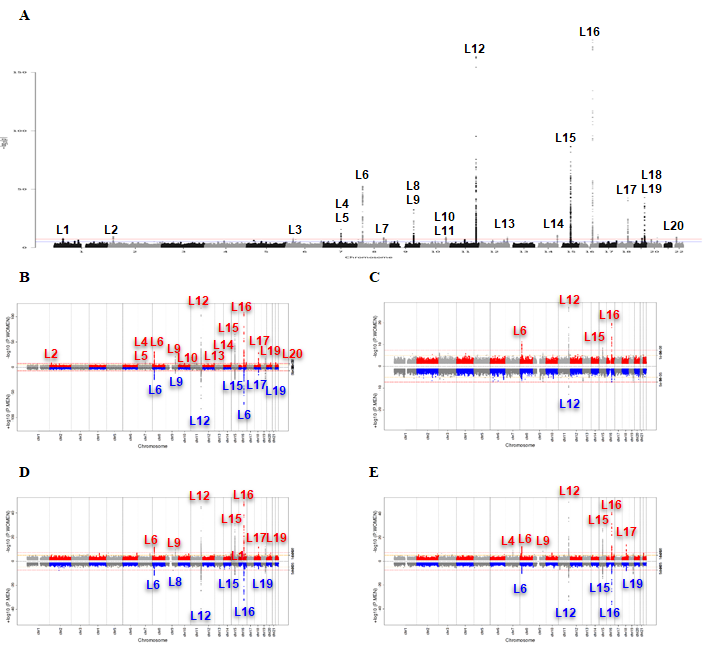

Supplement: Supplementary file 1 [file ijms-23-11889-s001.zip › Supplementary Figure S7. Manhattan & Maimi plot on HDL.tif]

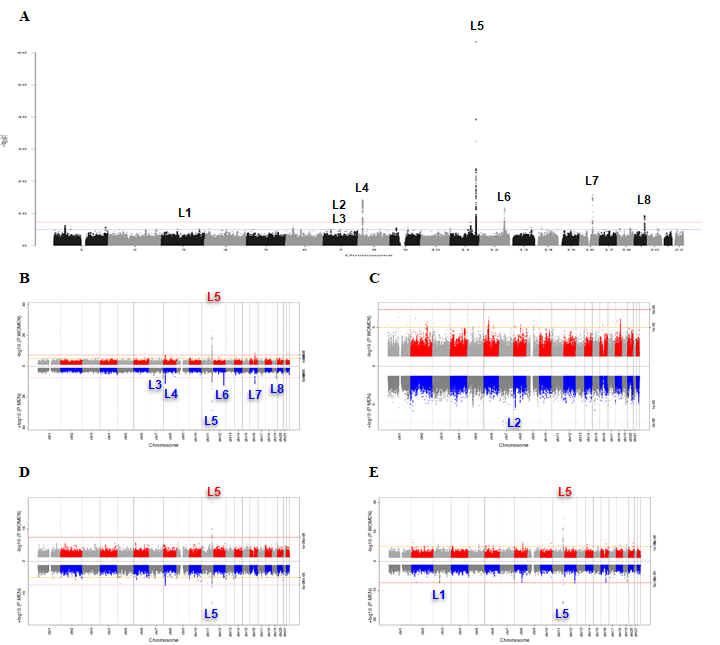

Supplement: Supplementary file 1 [file ijms-23-11889-s001.zip › Supplementary Figure S8. Manhattan & Maimi plot on MetS.tif]

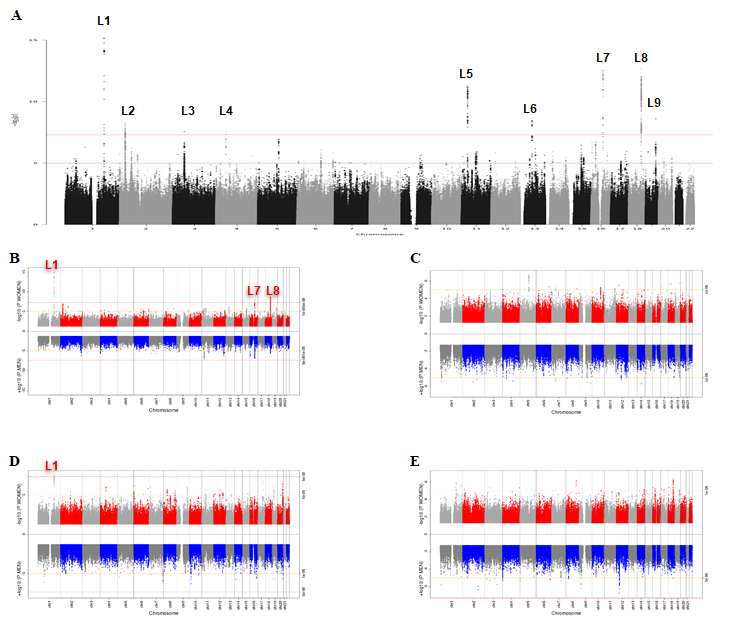

Supplement: Supplementary file 1 [file ijms-23-11889-s001.zip › Supplementary Figure S1. Manhattan & Maimi plot on BMI.tif]

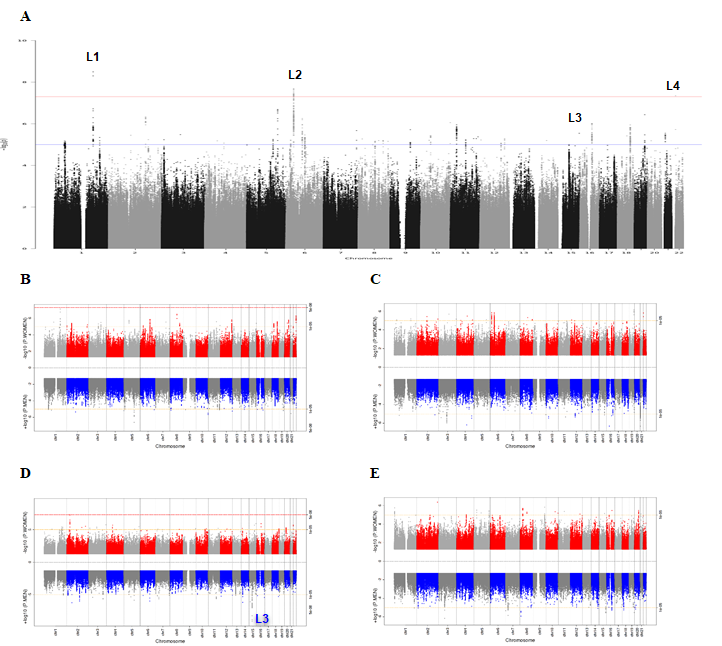

Supplement: Supplementary file 1 [file ijms-23-11889-s001.zip › Supplementary Figure S2. Manhattan & Maimi plot on Waist circ.tif]
